# Antarctic yeasts: analysis of their freeze-thaw tolerance and production of antifreeze proteins, fatty acids and ergosterol

**DOI:** 10.1186/s12866-018-1214-8

**Published:** 2018-07-05

**Authors:** Pablo Villarreal, Mario Carrasco, Salvador Barahona, Jennifer Alcaíno, Víctor Cifuentes, Marcelo Baeza

**Affiliations:** Departamento de Ciencias Ecológicas, Facultad de Ciencias, Universidad de Chile, Santiago, Chile

**Keywords:** Antifreeze proteins, Polyunsaturated fatty acids, Ergosterol, Antarctic yeasts, Freeze-thaw tolerance

## Abstract

**Background:**

Microorganisms have evolved a number of mechanisms to thrive in cold environments, including the production of antifreeze proteins, high levels of polyunsaturated fatty acids, and ergosterol. In this work, several yeast species isolated from Antarctica were analyzed with respect to their freeze-thaw tolerance and production of the three abovementioned compounds, which may also have economic importance.

**Results:**

The freeze-thaw tolerance of yeasts was widely variable among species, and a clear correlation with the production of any of the abovementioned compounds was not observed. Antifreeze proteins that were partially purified from *Goffeauzyma gastrica* maintained their antifreeze activities after several freeze-thaw cycles. A relatively high volumetric production of ergosterol was observed in the yeasts *Vishniacozyma victoriae*, *G. gastrica* and *Leucosporidium creatinivorum*, i.e., 19, 19 and 16 mg l^− 1^, respectively. In addition, a high percentage of linoleic acid with respect to total fatty acids was observed in *V. victoriae* (10%), *Wickerhamomyces anomalus* (12%) and *G. gastrica* (13%), and a high percentage of alpha linoleic acid was observed in *L. creatinivorum* (3.3%).

**Conclusions:**

Given these results, the abovementioned yeasts are good candidates to be evaluated for use in the production of antifreeze proteins, fatty acids, and ergosterol at the industrial scale.

**Electronic supplementary material:**

The online version of this article (10.1186/s12866-018-1214-8) contains supplementary material, which is available to authorized users.

## Background

Currently, there is a well-established and increasing global market for biomolecules used in industrial, medical, and biotechnological fields, such as antifreeze compounds, polyunsaturated fatty acids (PUFAs) and ergosterol. Among the antifreeze compounds, antifreeze proteins (AFPs) and ice-binding proteins (IBPs) have great biotechnological potential in the cryopreservation of mammalian and plant cells [1], preparation of frozen food and the cryopreservation of transplant organs [2, 3]. AFPs were first described almost four decades ago in Antarctic marine fishes [4] and have subsequently been discovered in a broad range of organisms, including snow mold fungi [5], sea ice diatoms [6], snow algae [7], bacteria [8–11] and yeasts [12–14]. AFPs are a large, non-homologous protein family with diverse structures [15, 16] and a common ability to bind to ice and modify its morphology and to inhibit ice recrystallization [17, 18], reducing cell injury due to ice formation. The isolation and purification of AFPs are laborious and costly processes, which limits their use at industrial scales [3, 18]. Thus, there has been a continuous search for cheaper sources of AFPs.

PUFAs are amphipathic molecules that have essential biological functions, such as the maintenance of cell membrane fluidity and permeability and enzyme activity, among others functions [19, 20]. Furthermore, the importance of PUFAs in the adaptation of organisms that inhabit cold environments or in the response to cold stress has been demonstrated [21–24]. PUFAs have also gained attention due to their roles in human health, therapeutics, and nutrition [25–27], and PUFAs are currently commercially obtained from plant seeds and some marine sources [25, 26]. Sterols are essential lipids in most eukaryotic cells that have important structural and signaling functions, with cholesterol, phytosterol, and ergosterol being the primary sterols present in vertebrates, plants and fungi, respectively [28]. Ergosterol is important economically, mainly because it is a precursor of vitamin D2 and has the potential for development of anticancer drugs since it inhibits the growth of different human cancer cell lines in vitro [29–32]. Thus, efforts have been made to improve the production of ergosterol in the yeast *Saccharomyces cerevisiae* for large-scale production [33, 34].

Due to the broad spectrum of applications for antifreeze compounds, ergosterol, and PUFAs, there has been a continuous search for attractive and novel sources of these products for their commercial production, such as microorganisms. Microorganisms have high growth rates and simple nutritional requirements. In addition, the ability to genetically manipulate and to perform large-scale fermentations with microorganisms makes them attractive model organisms. Among microorganisms, yeasts that thrive in extreme cold environments are of special interest because they naturally produce the previously mentioned compounds as part of their adaptive mechanisms to cold and freezing conditions [35, 36]. Because Antarctica has one of the driest and coldest climates on Earth, yeasts that thrive in Antarctica are good candidate sources of the previously mentioned compounds.

In previous studies, we reported the isolation and characterization of yeast species from different terrestrial habitats of Antarctica, focusing on the production of economically attractive hydrolytic enzymes and compounds, such as carotenoid pigments and mycosporines [37–43]. In this work, we analyzed the production of extracellular AFPs, PUFAs, and ergosterol in these Antarctic yeast species. For each type of compound, a potentially suitable yeast source was identified that could be a good candidate for further studies to evaluate its potential use in the commercial production of these compounds.

## Methods

### Yeasts and culture conditions

The yeast species used in this work are listed in Table 1 and were isolated and identified from soil and water samples from King George Island in the sub-Antarctic region [43]. The culture media used in this study were as follows: yeast-malt medium (YM), 0.3% yeast extract, 0.3% malt extract, 0.5% peptone; yeast nitrogen base (YNB), 0.67% yeast nitrogen base without amino acids, 0.5% peptone; Vogel’s minimal medium (VM), 13% Na_3_ citrate·2H_2_O, 12.6% KNO_3_, 14.4% (NH_4_)H_2_PO_4_, 8% KH_2_PO_4_, 1% MgSO_4_·7H_2_O, 10 μl trace element solution, and 5 μl 0.1 mg ml^− 1^ biotin solution. The media were supplemented with 2% glucose and contained 1.5% agar when agar-solidified medium was used. The yeasts were grown at various temperatures according to the optimal growth temperature for each species (Table 1).

**Table 1:** Yeast species used in this work

Species	T (°C)
*Candida parapsilosis*	30
*Candida sake*	22
*Cryptococcus gastricus (Goffeauzyma gastrica)*	22
*Cryptococcus gilvescens (Goffeauzyma gilvescens)*	22
*Cryptococcus victoriae (Vishniacozyma victoriae)*	22
*Dioszegia fristingensis*	22
*Leucosporidiella creatinivora (Leucosporidium creatinivorum)*	22
*Leucosporidiella fragaria (Leucosporidium fragarium)*	22
*Metschnikowia bicuspidata*	10
*Mrakia blollopis*	15
*Mrakia gelida*	22
*Mrakia sp.*	22
*Rhodotorula glacialis (Phenoliferia glacialis) (T11Rs)*	22
*Rhodotorula glacialis (Phenoliferia glacialis) (T8Rg)*	22
*Rhodotorula laryngis (Cystobasidium laryngis)*	22
*Rhodotorula mucilaginosa*	30
*Sporidiobolus salmonicolor*	22
*Wickerhamomyces anomalus*	30

The current taxonomic classification is given in parenthesis. T, best temperature for growth

### Tolerance of yeast to freeze-thaw cycles

The yeast strains were grown until the late log-phase of growth in YM medium supplemented with 2% glucose, after which 15 ml of each culture was aliquoted in separate falcon tubes. Each freeze-thaw cycle (FTC) consisted of freezing the culture aliquot in the falcon tube at − 20 °C for 12 h, after which it was thawed at 22 °C for 30 min. Sample aliquots were collected after each FTC, and the number of viable cells remaining was determined. The percentage of yeast survival was calculated by comparison to the number of viable cells in the original culture.

### Extraction and purification of secreted proteins

Yeast cultures (100 ml) were centrifuged at 8000 g for 10 min at 4 °C, after which the supernatants were filtered through a sterile 0.45-μm pore size polyvinylidene fluoride membrane (Millipore, Billerica, MA, USA). Then, ammonium sulfate was added stepwise to cell-free supernatants to reach 80% saturation, followed by incubation at 4 °C for 2 h with orbital agitation. The protein pellets obtained after centrifugation at 10,000 g at 4 °C for 10 min were suspended in 5 ml of water and dialyzed against 1000 ml of water for 12 h with 2 changes of water using a dialysis bag with a 10-kDa cut-off. The same procedure was performed for each step of sample fractioning by ammonium sulfate precipitation from 20 to 80% saturation. For chromatographic purification, a Superdex 75 10/300 GL column (Merck, Darmstadt, Germany) equilibrated with 20 mM sodium phosphate buffer (pH 7.0) and 150 mM NaCl was used as the mobile phase with a flow rate of 0.2 ml min^− 1^. The chromatographic runs were performed using an AKTA prime purification system (General Electric, New York, USA), with protein elution monitored at 280 nm, and 0.2-ml fractions were collected. Fractions corresponding to protein peaks were dialyzed as described above and were tested for antifreeze activities. The protein profile was determined by 15% SDS-PAGE, and proteins were quantified using a BCA kit (Pierce BCA protein assay kit, Thermo Scientific, IL, USA) according to the manufacturer’s instructions. To assess protein glycosylation, the SDS-PAGE gels were stained using a Pierce glycoprotein staining kit (Thermo Scientific, IL, USA) according to the manufacturer’s instructions.

### Evaluation of the antifreeze properties of protein samples

The antifreeze properties of protein samples were evaluated by determining the inhibition of ice recrystallization using a method based on the aggregation of gold nanoparticles [44, 45]. Briefly, gold nanoparticles (AuNP) were synthesized by heating 300 mM HAuCl_4_·3H_2_O until it began to boil, after which 2 ml of 30 mM sodium citrate dehydrate was added and the solution was boiled for 20 min and then cooled to ambient temperature with continuous stirring. Next, one volume of 2-mercaptosuccinic acid was added, and the mixture was agitated for 1 h at ambient temperature. To assess the antifreeze properties of protein samples, the AuNP solution and a protein sample (50 μl each) were mixed in one well of a microtiter plate, which was frozen at − 20 °C for 1 h and then thawed at 22 °C for 30 min. The absorbance spectrum from 400 to 800 nm was recorded using an Epoch microplate spectrophotometer (Biotek, Winooski, VT, USA) before and after freezing. The A_520_/A_650_ ratio was calculated before (BFR) and after (AFR) the freezing step, and the BFR/AFR ratio was determined for each sample. The closer the BFR/AFR value is to one, the greater the antifreeze activity of the tested sample.

### Sterol extraction and identification

Sterols were extracted using the method described by Shang et al. [46]. First, the cell pellet from 10 ml of yeast culture was mixed with 4 g of KOH and 16 ml of 60% ethanol. After incubation at 80 °C for 2 h, sterols were extracted with 10 ml of petroleum ether and quantified at 282 nm using a molar extinction coefficient of 11,900 cm^− 1^ M^− 1^ [47]. For the reverse phase high-pressure liquid chromatography (RP-HPLC) analysis, samples were dried at 25 °C and suspended in 200 μl acetone. Samples were then loaded onto a LiChroCART RP18 125–4 column (Merck KGaA, Darmstadt, Germany) using methanol:water (97:3, *v*/v) as the mobile phase at a 1.8 ml min^− 1^ flow rate. Analyses were performed in an LC-10ATVP Shimadzu instrument equipped with a diode array detector, and sterols were detected at 280 nm.

### Analysis of fatty acid composition

Yeasts were cultured in 8 l of YM medium supplemented with 1% glucose in a BIOFLO 415 fermenter (New Brunswick Scientific, Edison, NJ, USA) until the stationary phase of growth was reached. Cell pellets were collected by centrifugation at 4000 g at 4 °C for 5 min and were thoroughly washed with distilled water. Oil extraction and analysis were conducted according to the method described by Bligh and Dyer [48] and the AOAC official method 969.33, which were performed by an external service (ANALAB CHILE S.A., www.analab.cl).

## Results

### Tolerance to freeze-thaw cycles

**Fig. 1:**
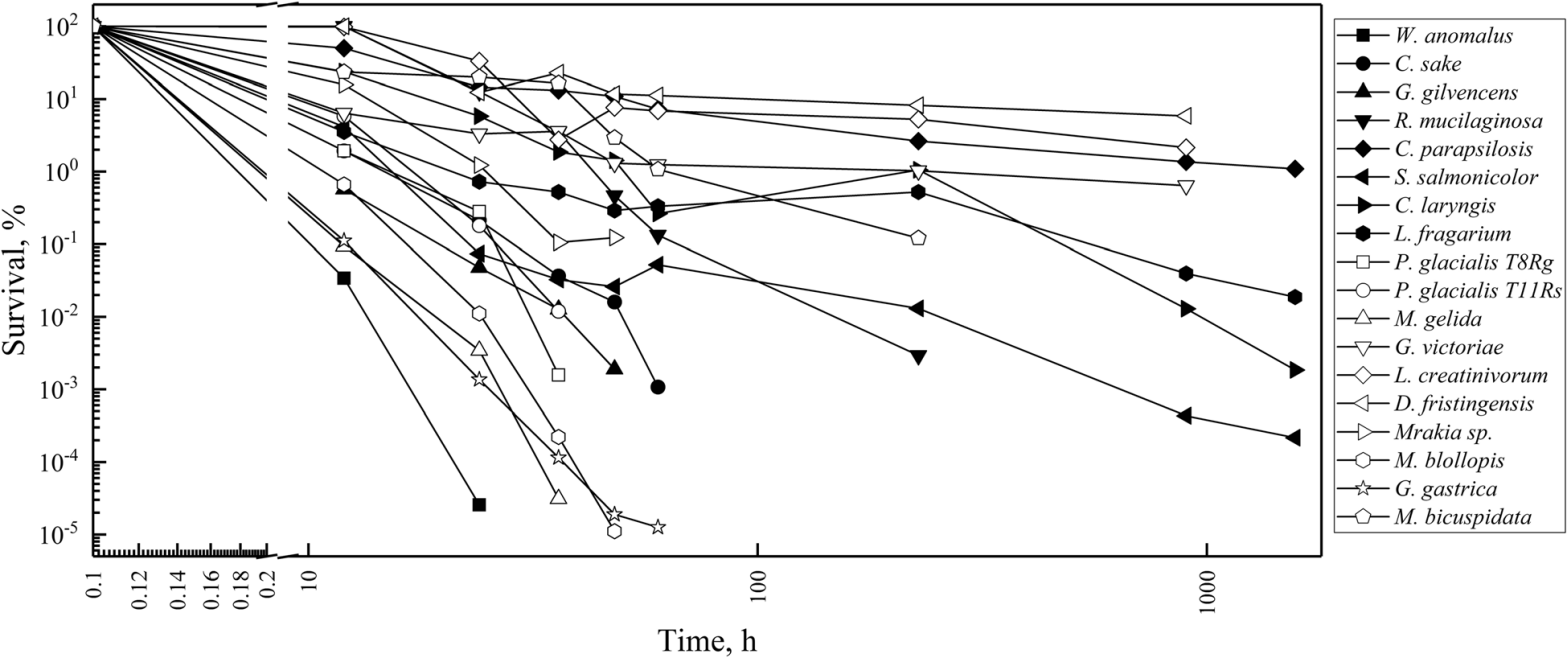
Tolerance to freeze-thaw cycles. Yeast cultures were frozen at − 20 °C and then thawed at 22 °C. The freezing time varied according to the results obtained for each yeast species in successive FTCs. The values are the average of three determinations. The error bars were not included to maintain the clarity of the plot

Yeast cultures were subjected to FTCs as described in the Materials and Methods, although the freezing time varied for some yeast species that showed no loss of survival between successive FTCs. As shown in Fig. 1, the freeze-thaw tolerance varied among the studied yeasts species: *Dioszegia fristingensis*, *Leucosporidium creatinivorum, Candida parapsilosis*, and *Vishniacozyma victoriae* exhibited the highest tolerance, displaying survival percentages of 8, 5 3, and 1%, respectively, after six FTCs. By contrast, the yeasts *Wickerhamomyces anomalus*, *Goffeauzyma gastrica*, *Mrakia gelida*, and *Mrakia blollopis* showed the lowest freeze-thaw tolerance, as less than 0.1% of cells survived after two FTCs. The remainder of the assayed yeast species showed moderate freeze-thaw tolerance compared to those mentioned above. For comparative purposes, some yeasts with high, moderate and low freeze-thaw tolerance were selected for further study.

In the previous assay, yeasts were cultivated at their optimal growth temperature before being submitted to FTCs. We then evaluated whether incubation at a lower temperature prior to FTCs would improve their freeze-thaw tolerance. To test this possibility, *L. creatinivorum* and *G. gastrica*, species with high and low freeze-thaw tolerances, respectively, were cultured at their optimal growth temperature until the late exponential phase of growth was reached. Next, the yeasts were incubated at 4 °C for 24, 48 and 72 h before being subjected to FTCs. No freeze-thaw tolerance improvement was observed for either strain in all assays performed (data not shown).

### Evaluation of the antifreeze properties of proteins secreted by yeasts and their purification

Total extracellular proteins were obtained from cell-free supernatants of yeast cultures, after which they were analyzed by SDS-PAGE and assayed for their antifreeze properties (Additional file 1: Figure S1). The BFR/AFR ratio was also determined for 1 μg ml^− 1^ bovine serum albumin (BSA) (used as the control) and for protein samples extracted from non-inoculated YM medium, which had values of 0.24 and 0.51, respectively. Protein samples with a BFR/AFR ratio higher than those observed for BSA and non-inoculated YM medium were obtained from *L. creatinivorum* (0.82), *C. parapsilosis* (0.94) and *G. gastrica* (0.91). The components of YM medium such as proteins and pigments co-precipitated when the culture supernatant protein samples were obtained, which could influence the determination of antifreeze properties. For this reason, the yeast biomass and extracellular protein yields of the three yeast species mentioned above were compared when cultivated in YM medium and the less complex Vogel, YNB, and YNB-P media. Although the results obtained using the last three media were lower than those obtained using the YM medium (Additional file 2: Table S1), the best results were obtained using the YNB-P medium supplemented with 2% glucose for all three yeast species. Therefore, YNB-P supplemented with 2% glucose was selected for further protein purification steps and analysis.

**Fig. 2:**
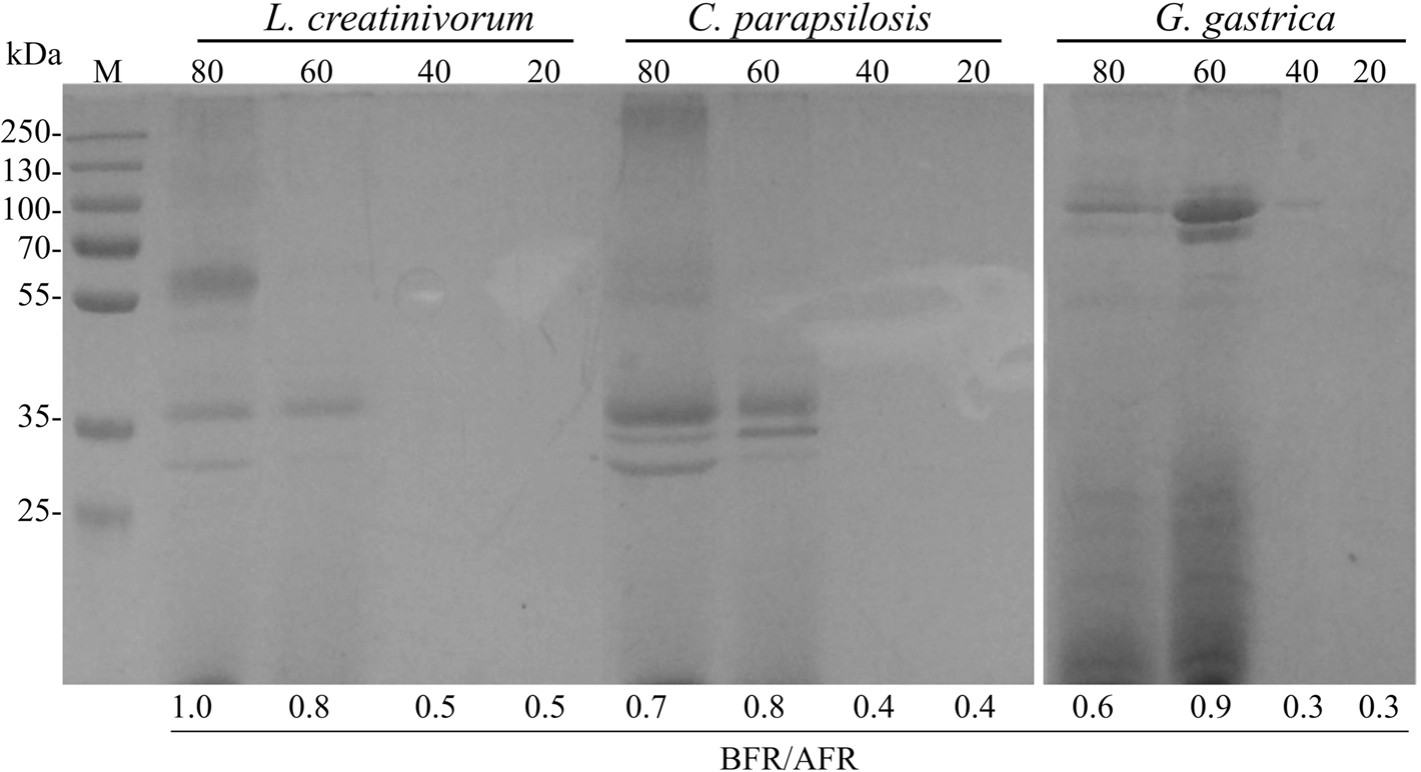
Fractioning and antifreeze properties of proteins secreted by yeasts. The different yeast species were cultured in YNB-P medium until the stationary growth phase. The proteins were fractionated from cell-free supernatants using increasing saturation percentages of ammonium sulfate (indicated at the top of the SDS-PAGE gels). The antifreeze activity (BFR/AFR) of each protein fraction is indicated at the bottom of each gel well. M, protein molecular marker

**Fig. 3:**
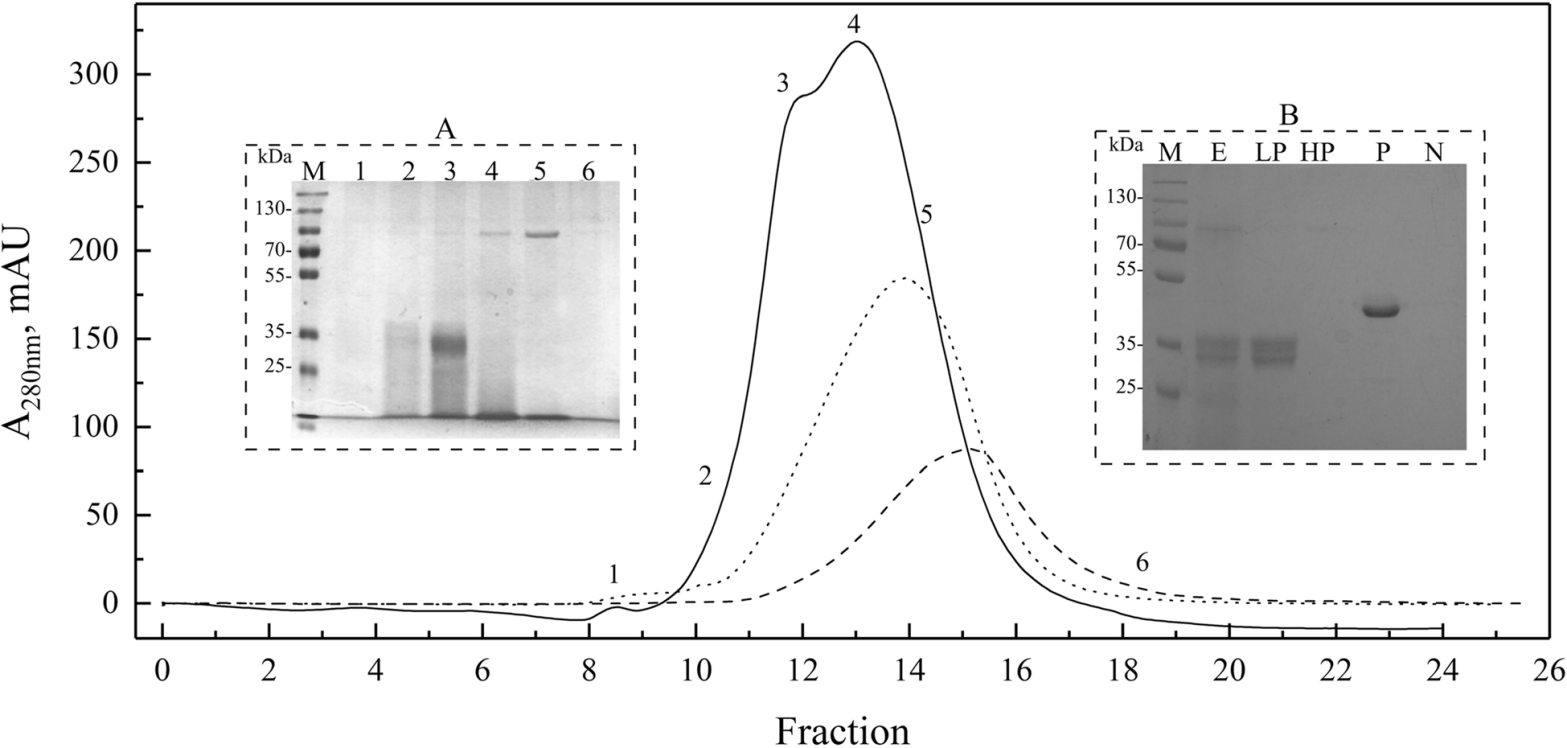
Chromatographic separation of proteins from yeast samples. The sample corresponding to the ammonium sulfate fraction with the highest antifreeze activity from *L. creatinivorum* (dotted line), *C. parapsilosis* (discontinuous line) and *G. gastrica* (continuous line) was loaded onto a Superdex 75 10/300 GL column. The mobile phase consisted of 20 mM sodium phosphate buffer at pH 7.0 and 150 mM NaCl at 0.2 ml min^−1^. A, SDS-PAGE analysis of fractions numbered from 1 to 5 (indicated in the corresponding chromatogram) from *G. gastrica*. B, Analysis of protein glycosylation: E, original extract; LP, low-molecular-weight proteins; HP, high-molecular-weight proteins; P, positive control; N, negative control. M, protein marker

**Fig. 4:**
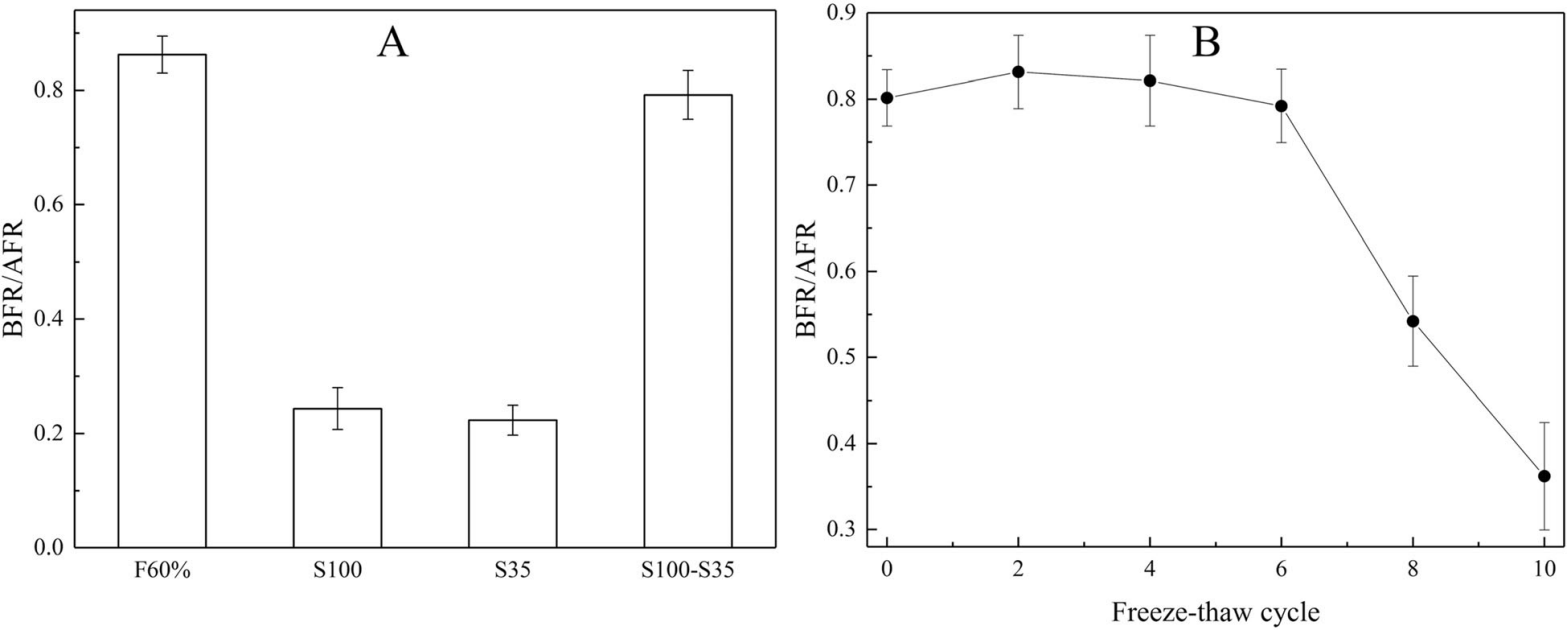
Freeze-thaw tolerance of proteins from *G. gastrica*. A, antifreeze properties of protein samples corresponding to fractions: 60% ammonium sulfate (F60%), rMW 100,000 (S100), rMW lower than 35,000 (S35) and a mix of S100 and S35 (S100-S35). B, antifreeze properties of S100-S35 determined after several freeze-thaw cycles. The values are the average of three determinations, and the error bars correspond to the standard deviation

Figure 2 shows the protein content (≥10 kDa according to the cut-off of the dialysis bag used in the purifications) and antifreeze activity analyses of protein samples fractionated using different concentrations of ammonium sulfate. The highest BFR/AFR values were observed for the protein fractions obtained from the *L. creatinivorum*, *C. parapsilosis* and *G. gastrica* protein samples at 80, 60 and 60% ammonium sulfate saturation, respectively. These fractions were subjected to additional purification steps using several chromatographic methods, and the best results were obtained using cationic exchange chromatography (Fig. 3). A protein band with an rMW of approximately 100,000 (S100) and two other bands with an rMW less than 35,000 (S35) (box A in Fig. 3) could be distinguished in the protein samples from *G. gastrica*, all of which are glycosylated proteins (box B in Fig. 3). For the protein samples from the yeasts *L. creatinivorum* and *C. parapsilosis*, no clear protein bands were observed (data not shown). Interestingly, no antifreeze activity could be detected when the protein samples containing the S100 or the S35 protein bands from *G. gastrica* were assayed independently. However, the antifreeze activity was restored and reached a similar level as the original protein sample (the 60% ammonium sulfate saturation fraction) when fractions containing each protein band were mixed (Fig. 4a). The protein mix was subjected to several FTCs and assessed for antifreeze activity after each cycle. As shown in Fig. 4b, the protein mix maintained its antifreeze activity until the sixth FTC but decreased rapidly after additional cycles.

### Fatty acids

The results from the fatty acid analyses are summarized in Table 2, including the composition details for PUFAs. Details for the composition of saturated and monounsaturated FAs are included in Additional file 3: Table S2. Among the identified monounsaturated FAs, higher percentages of oleic acid (C18:1) were observed in *Dioszegia fristingensis* (57%), *Rhodotorula mucilaginosa* (57%) and *Cystobasidium laryngis* (63%), corresponding to 35, 34 and 42% of their total FAs, respectively. Eicosanoic acid (EPA, C20:1) was present at high percentages in *R. mucilaginosa* (1.7%) and *C. laryngis* (2.1%), representing 1 and 1.4% of total FAs, respectively. With respect to PUFAs, the highest percentages, from 30 to 37%, were observed in the yeasts *W. anomalus*, *G. gastrica*, *V. victoriae* and *L. creatinivorum*. Among PUFAs, the highest percentages of linoleic acid (LA, C18:2) were observed in *G. victoriae* (30%), *W. anomalus* (33%) and *G. gastrica* (35%), representing 10, 12 and 13% of total FAs, respectively. The highest percentage of alpha linoleic acid (ALA, 18:3) was observed in *L. creatinivorum* (11%), corresponding to 3.3% of total FAs.

### Extraction and identification of sterols

**Fig. 5:**
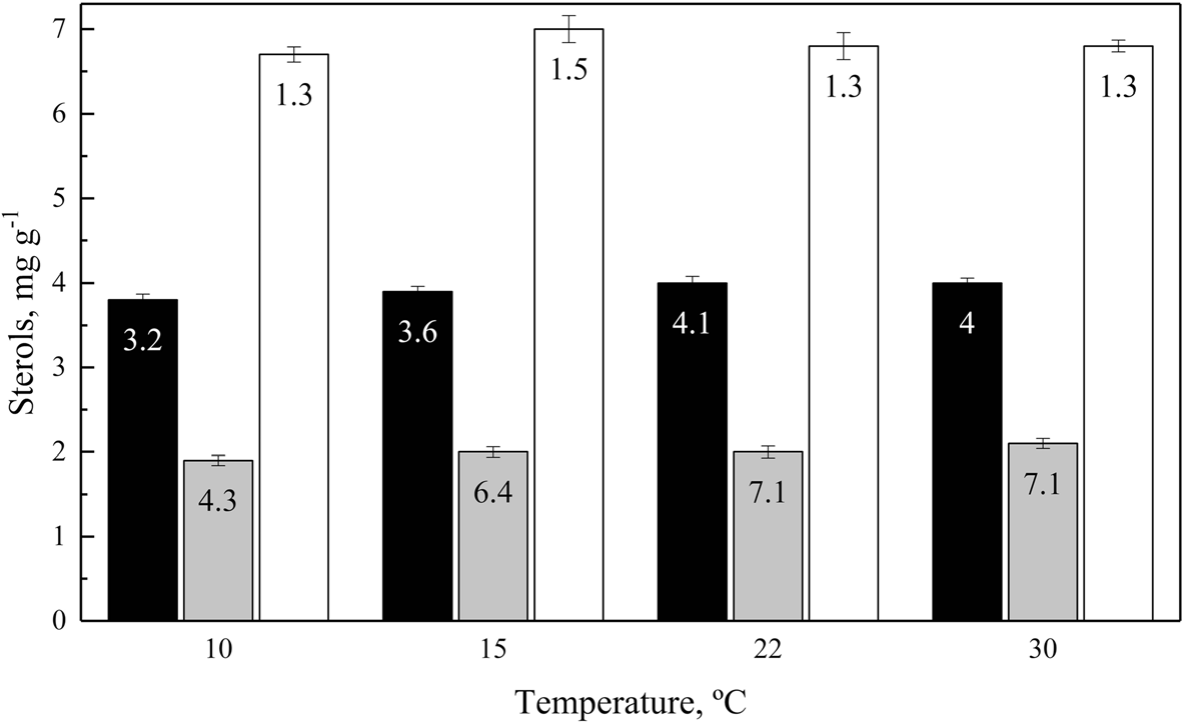
Sterol contents in yeasts cultivated at different temperatures. The yeasts *M. blollopis* (white columns), *L. creatinivorum* (black columns), and *R. mucilaginosa* (grey columns) were grown until the early stationary phase of growth, and sterols were extracted and quantified from the cell pellets. The biomass (in g l^− 1^) reached by each culture at each assayed temperature is indicated in each column. The values are the average of three determinations, and the error bars correspond to the standard deviation

The yeast species were grown in YM medium supplemented with glucose at their optimal growth temperatures until the stationary phase of growth, and yeast pellets were obtained. Sterols were extracted from the pelleted yeast cells and were quantified and analyzed by RP-HPLC. The yeasts with the highest sterol contents (higher than 3 mg g^− 1^ dry weight) were *M. blollopis*, *L. creatinivorum*, *D. fristingensis* and *M. gelida* (Table 3). The effect of cultivation temperature on the production of sterols was evaluated from 10 to 30 °C for yeasts with high (*M. blollopis* and *L. creatinivorum)* and low (*R. mucilaginosa*) sterol contents. No significant changes in total sterol contents for either yeast were observed when they were cultivated at the different temperatures (Fig. 5).

**Table 2:** Fatty acids composition in Antarctic yeasts

Yeast species	Fatty acids, %					
	Saturated	Monounsaturated	Polyunsaturated			
			Total	LA(C18:2)	ALA(C18:3)	C20:2 <
*L. creatinivorum*	23.1	42.7	30.2	18.6	10.8	0.8
*C. parapsilosis*	44.9	20.0	16.8	13.5	1.9	1.4
*V. victoriae*	37.3	29.6	33.1	30.1	2.2	0.8
*D. fristingensis*	11.0	60.9	28.1	21.8	6.3	ND
*R. mucilaginosa*	15.5	60.0	20.6	17.8	2.2	0.6
*C. laryngis*	20.2	67.1	12.1	11.5	nd	0.6
*S. salmonicolor*	43.7	41.9	14.4	14.4	nd	nd
*W. anomalus*	21.9	37.3	36.7	33.1	3.1	0.5
*G. gastrica*	13.3	43.3	36.4	34.9	nd	1.5
*M. gelida*	49.3	38.1	12.6	12.6	nd	nd
*M. blollopis*	44.0	29.7	26.3	24.5	0.7	1.2

*nd* not detected, *LA(C18:2)* linoleic acid, *ALA(C18:3)* alpha linolenic acid, *C20:2 <* PUFAs of 20 carbons with more than two unsaturation

**Table 3:** Analysis of sterols content in different yeast species

Yeast species	Biomass	Total^a^, mg g^−1^
*L. creatinivorum*	4.0 ± 0.1	4.03 ± 0.12*
*C. parapsilosis*	8.2 ± 1.0	1.94 ± 0.17
*V. victoriae*	12.6 ± 2.4	1.52 ± 0.02
*D. fristingensis*	3.6 ± 0.2	3.43 ± 0.25
*R. mucilaginosa*	5.8 ± 0.3	2.07 ± 0.04*
*C. laryngis*	6.5 ± 0.3	2.44 ± 0.09*
*S. salmonicolor*	5.0 ± 0.6	2.63 ± 0.13
*W. anomalus*	7.1 ± 0.2	2.23 ± 0.08*
*G. gastrica*	8.7 ± 0.8	2.13 ± 0.22*
*M. gelida*	3.2 ± 0.2	3.11 ± 0.02
*M. blollopis*	1.4 ± 0.1	6.91 ± 0.24

^a^, normalized by dry weight of yeasts; ^b^, percentages calculated according to analysis by RP-HPLC. *, data from Villarreal et al. [38]. The values are the average of three determinations

The sterol composition of the different yeast species was analyzed by RP-HPLC. In all cases, a major peak was observed that corresponded to ergosterol according to the retention time and absorbance spectrum (Additional file 4: Figure S2). These results indicate that *M. blollopis* and *L. creatinivorum* had the highest ergosterol content by yeast dry weight among the assayed strains, i.e., 6.9 and 4.0 mg g^− 1^, respectively. However, considering the culture volume, the major ergosterol producers were *V. victoriae*, *G. gastrica* and *L. creatinivorum*, which produced 19.2, 18.5 and 16.1 mg l^− 1^ of ergosterol, respectively.

## Discussion

In this work, yeast species isolated from Antarctica were analyzed for the production of ice-binding proteins, unsaturated fatty acids and ergosterol, compounds associated with tolerance to cold and freezing conditions and that are economically attractive. From a physiological perspective, none of these metabolites alone could be correlated with the tolerance to freeze-thaw cycles performed in this study (Additional file 5: Figure S3), suggesting that there is a complex response to this stress that may involve other factors not analyzed in this study, such as the production of intracellular IBPs and compatible metabolites.

Antifreeze activity was detected in the secreted proteins of *L. creatinivorum, C. parapsilosis,* and *G. gastrica*. However, it is important to mention that if some of the studied yeasts secreted AFPs with a minor molecular weight ≥ 10 kDa, these could not be detected due to the purification methodology used in this study. In spite of this, our work increases the number of yeast species from which AFPs or antifreeze activity has been reported, which so far have been described only for *Glaciozyma antarctica*, *Glaciozyma* sp. AY30 (formerly *Leucosporidium* sp. AY30), and *Rhodotorula glacialis* [49]. AFPs were partially purified only from *G. gastrica*, in which at least three proteins with rMWs of 30,000 to 100,000 are responsible for this activity. The majority of antifreeze proteins described so far are small (less than 25 kDa) [15], and to the best of our knowledge, there are no reports describing several proteins acting together to produce antifreeze activity. These proteins maintained their antifreeze activity after several FTCs, a desirable characteristic for potential applications as additives for preserving the viability of frozen cells. The most studied IBP is a 26-kDa glycosylated protein from an Artic *Glaciozyma* sp. [50], and its application in preserving several types of eukaryotic cells, including erythrocytes and mouse oocytes, has been documented [12, 51–53]. Commercially, natural and synthetic AFPs are expensive, and studies on those expressed in bacteria, yeast and insect cell culture systems are necessary for more efficient expression and scale-up production [54]. Furthermore, there is a constant search for IBPs with new properties for application in different fields, for which more in-depth characterization of the IBPs described here is necessary to evaluate their applicability.

One of the most studied adaptations of yeasts that live in cold environments is their lipid metabolism, which allows them to maintain membrane fluidity at low temperatures, an example of which is the increased production of unsaturated FAs [22]. In six of the Antarctic yeast species investigated in this study, the percentage of unsaturated FAs was higher than 70%, which is consistent with others studies and indicates that Antarctic yeasts produce a higher proportion of unsaturated FAs than yeasts isolated from other environments [55–57]. Oleic, eicosanoic, linoleic and alpha-linoleic acids, which are economically important FAs since they must be incorporated in mammalian diets [19, 58, 59], were all identified at high percentages in at least one of the yeast species analyzed in this work. Taking into account the total FAs, the percentages of PUFAs in the yeast species analyzed in this work ranged from 12 to 37%, which are very high values compared to the 4% obtained in a genetically engineered strain of *S. cerevisiae* [60]. Alternatives to current sources of PUFAs are desirable to lower the cost of production and to eliminate the fishy flavor of PUFAs-fortified foods. Yeasts have many attributes that support the economic feasibility of their large-scale production, such as high growth rates and production yields using inexpensive growth media, as well as the ability to increase their productivity through culture optimization and genetic manipulations [61–63]. Although lipid or PUFA synthesis has been achieved through genetic engineering, the culture conditions that favor lipid accumulation, the high synthesis of PUFAs and cell growth are lacking, which limits large-scale economic production [64].

In all yeast species analyzed in this study, almost 100% of sterols found corresponded to ergosterol. Although the highest specific content of ergosterol was observed for *M. blollopis*, *V. victoriae* produced the highest amount of ergosterol per liter of culture. The commercial sources of ergosterol correspond to strains of *S. cerevisiae*; however, the ergosterol content is finely regulated in this yeast species at the transcriptional level, resulting in a low production [65]. The ergosterol contents of *M. blollopis* and *L. creatinivorum* were comparable to a genetically modified strain of *S. cerevisiae* [33, 34], raising the possibility of future improvements of their natural ergosterol production at an industrial scale through the modification of culture conditions.

## Conclusions

From a physiological perspective, none of the compounds analyzed in this study may solely explain the freeze-thaw tolerance observed for the yeast species investigated, suggesting a complex response to this stress that may include other cellular mechanisms that were not considered. With respect to the production of economically important compounds, we identified yeasts that are good candidates for the industrial production of AFPs, PUFAs and ergosterol.

## Additional files

Additional file 1: **Figure S1.** SDS-PAGE results of the proteins secreted by yeasts and their antifreeze properties. The corresponding yeast species from which the proteins sample were obtained and their BFR/AFR values are indicated at the top and bottom of the wells, respectively. M, protein molecular marker. (JPG 951 kb)

Additional file 2: **Figure S2.** Representative RP-HPLC chromatograms from sterol sample analyses. Yeasts with the highest (*M. blollopis*, continuous line) and the lowest (*V. victoriae* discontinuous line) sterol contents were included. The absorbance spectra for the corresponding peaks are shown. (JPG 698 kb)

Additional file 3: **Figure S3.** Biplot of the two principal components derived from the biomolecular data. SFA, saturated fatty acids; MUFA, monounsaturated fatty acids; PUFA, polyunsaturated fatty acids; AP, antifreeze property; Erg, ergosterol. At each point, the tolerance to FTCs is indicated. In each case, the percentages were calculated considering the highest value as 100%. (JPG 761 kb)

Additional file 4: **Table S1.** Yeast growth and production of extracellular proteins in different media. (DOCX 50 kb)

Additional file 5: **Table S2.** Saturated and monounsaturated fatty acid composition in Antarctic yeasts. (DOCX 17 kb)

## Abbreviations

AFPs: Antifreeze proteins
AFR: The A_520_/A_650_ ratio after freezing
ALA: Alpha linoleic acid
AP: Antifreeze property
AuNPs: Gold nanoparticles
BFR: The A_520_/A_650_ ratio before freezing
BSA: Bovine serum albumin
FAs: Fatty acids
FTC: Freeze-thaw cycle
IBPs: Ice-binding proteins
LA: Linoleic acid
MUFAs: Monounsaturated fatty acids
PUFAs: Polyunsaturated fatty acids
RP-HPLC: Reverse-phase high performance liquid chromatography
SFAs: Saturated fatty acids
VM: Vogel’s minimal medium
YM: Yeast-malt medium
YNB: Yeast nitrogen base
