# Supplementary figures and images for: Antarctic yeasts: analysis of their freeze-thaw tolerance and production of antifreeze proteins, fatty acids and ergosterol
Source: BMC Microbiol. 2018 Jul 5;18:66. doi: 10.1186/s12866-018-1214-8 (PMC6034288; doi:10.1186/s12866-018-1214-8)

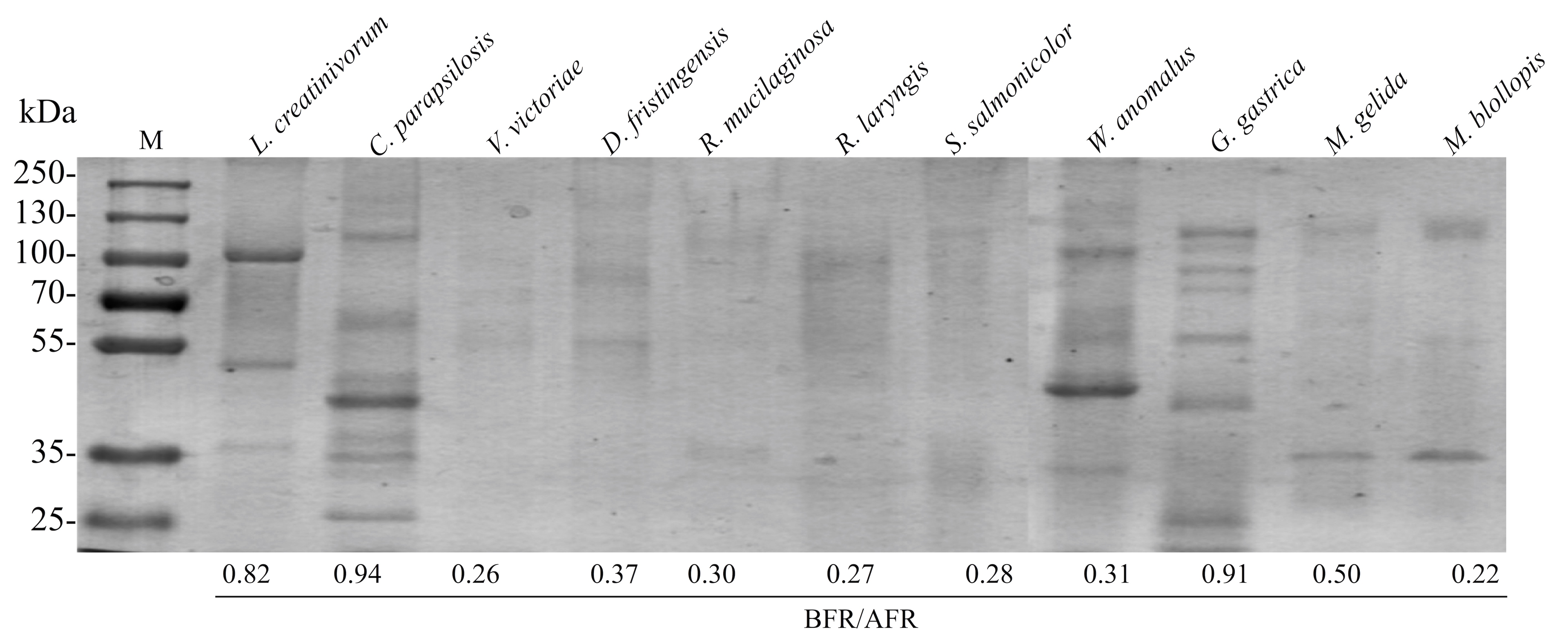

Supplement: Supplementary file 1 — Figure S1. SDS-PAGE results of the proteins secreted by yeasts and their antifreeze properties. The corresponding yeast species from which the proteins sample were obtained and their BFR/AFR values are indicated at the top and bottom of the wells, respectively. M, protein molecular marker. (JPG 951 kb) [file 12866_2018_1214_MOESM1_ESM.jpg]

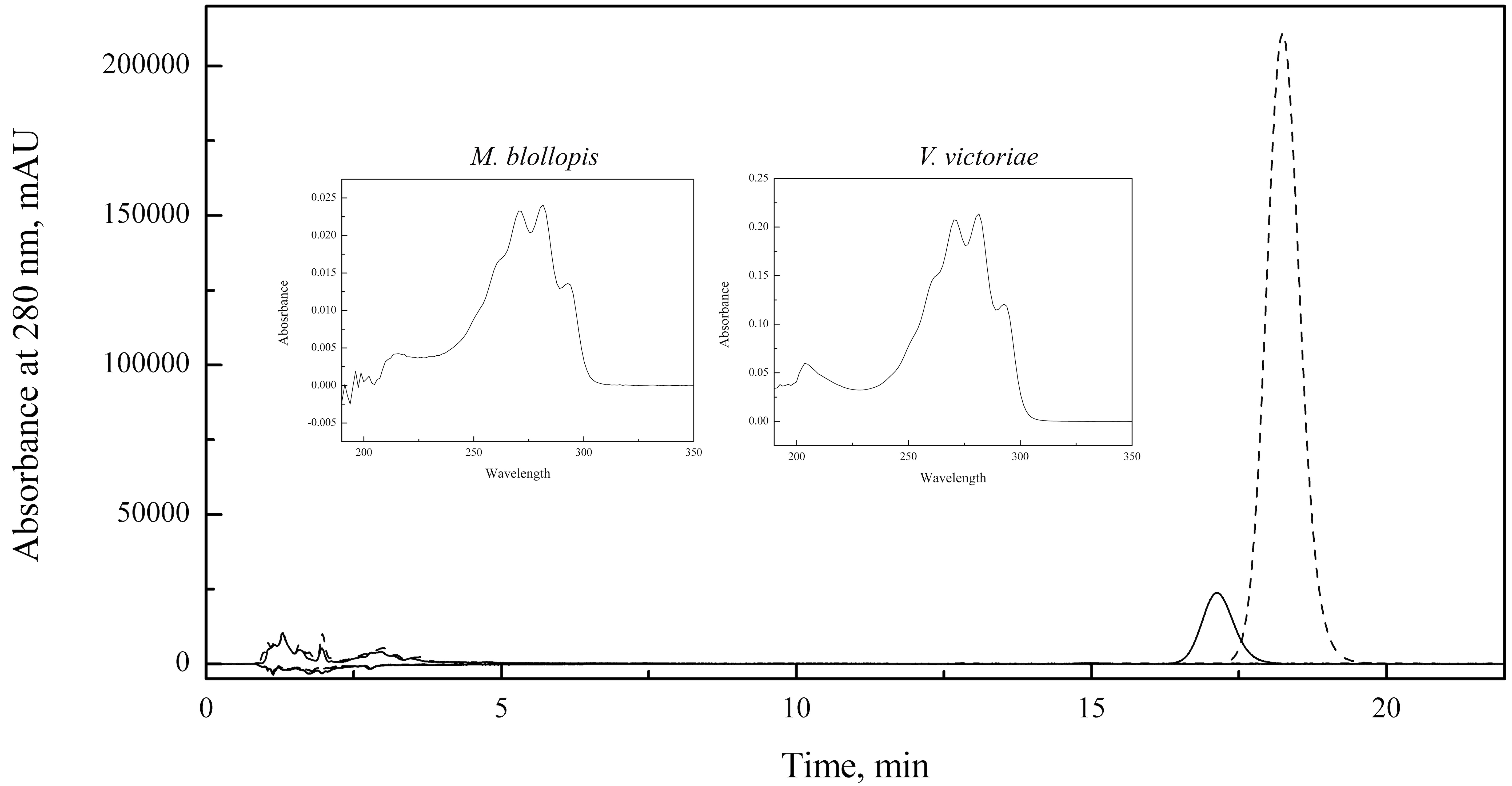

Supplement: Supplementary file 4 — Table S1. Yeast growth and production of extracellular proteins in different media. (DOCX 50 kb) [file 12866_2018_1214_MOESM4_ESM.jpg]

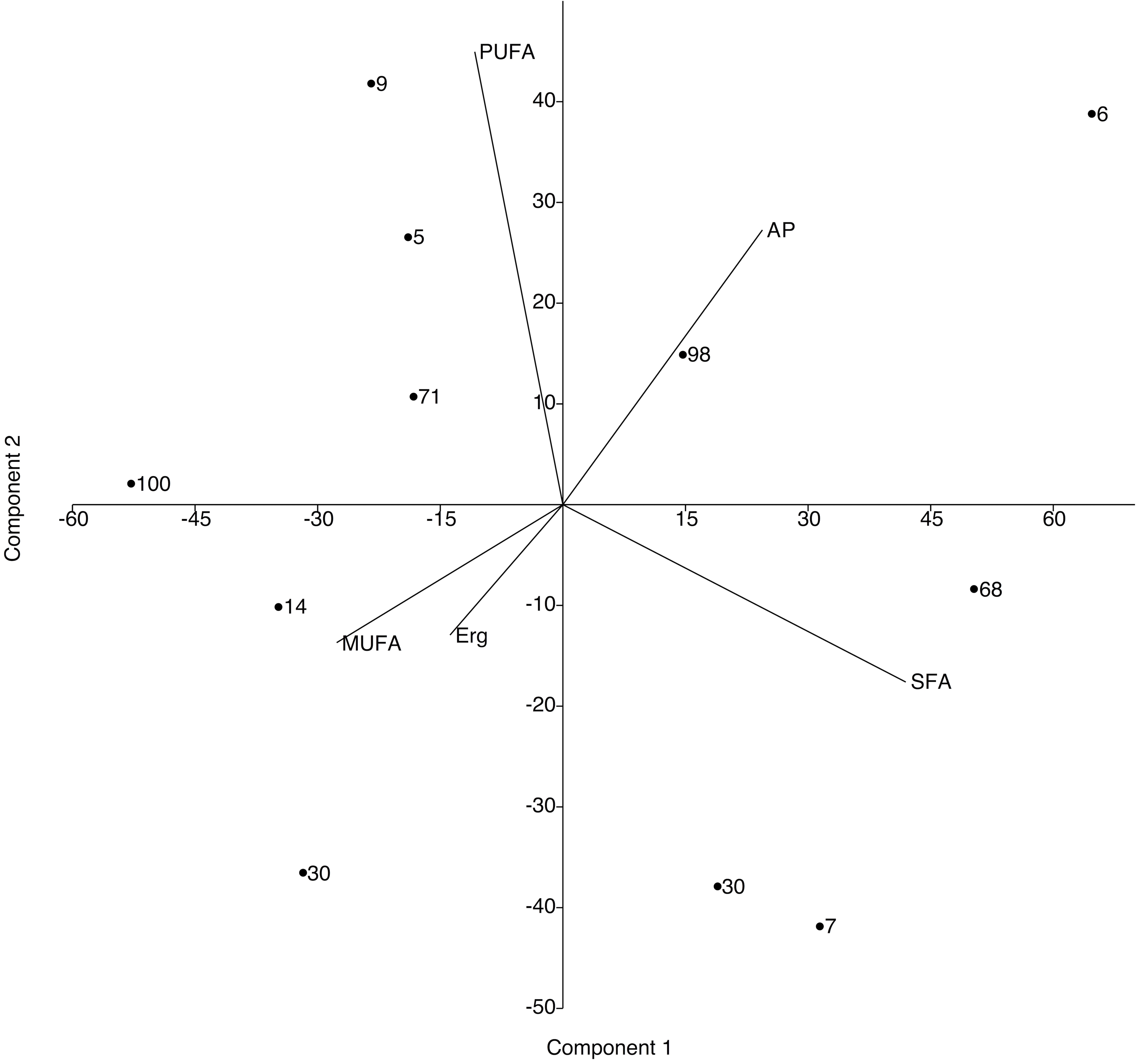

Supplement: Supplementary file 5 — Table S2. Saturated and monounsaturated fatty acid composition in Antarctic yeasts. (DOCX 17 kb) [file 12866_2018_1214_MOESM5_ESM.jpg]
