# Supplementary material for: Antarctic yeasts: analysis of their freeze-thaw tolerance and production of antifreeze proteins, fatty acids and ergosterol
Source: BMC Microbiol. 2018 Jul 5;18:66. doi: 10.1186/s12866-018-1214-8 (PMC6034288; doi:10.1186/s12866-018-1214-8)
Supplement: Supplementary file 2 — Figure S2. Representative RP-HPLC chromatograms from sterol sample analyses. Yeasts with the highest (M. blollopis, continuous line) and the lowest (V. victoriae discontinuous line) sterol contents were included. The absorbance spectra for the corresponding peaks are shown. (JPG 698 kb) [file 12866_2018_1214_MOESM2_ESM.docx]

**Table S1.** Yeast growth and production of extracellular proteins in different media.

| Species | Media | Biomass (g l^-1^)* | Proteins (μg ml^-1^) |
| --- | --- | --- | --- |
| *L. creatinivorum* | YM | 466 ± 10 | 3,010 ± 110 |
|  | V | 182 ± 6 | 210 ± 10 |
|  | YNB | 308 ± 7 | 1,010 ± 2 |
|  | YNB-P | 362 ± 2 | 2,810 ± 3 |
| *C. parapsilosis* | YM | 389 ± 3 | 3,220 ± 19 |
|  | V | 101 ± 4 | 1,300 ± 14 |
|  | YNB | 112 ± 2 | 1,580 ± 23 |
|  | YNB-P | 274 ± 11 | 2,390 ± 16 |
| *G. gastrica* | YM | 421 ± 8 | 4,830 ± 91 |
|  | V | 125 ± 5 | 670 ± 14 |
|  | YNB | 287 ± 9 | 910 ± 8 |
|  | YNB-P | 294 ± 7 | 2,660 ± 77 |

YM, yeast medium; V, vogel medium; YNB, yeast nitrogen base medium; YNB-P, YNB supplemented with peptone.*, wet weight.
