# Supplementary material for: Antarctic yeasts: analysis of their freeze-thaw tolerance and production of antifreeze proteins, fatty acids and ergosterol
Source: BMC Microbiol. 2018 Jul 5;18:66. doi: 10.1186/s12866-018-1214-8 (PMC6034288; doi:10.1186/s12866-018-1214-8)
Supplement: Supplementary file 3 — Figure S3. Biplot of the two principal components derived from the biomolecular data. SFA, saturated fatty acids; MUFA, monounsaturated fatty acids; PUFA, polyunsaturated fatty acids; AP, antifreeze property; Erg, ergosterol. At each point, the tolerance to FTCs is indicated. In each case, the percentages were calculated considering the highest value as 100%. (JPG 761 kb) [file 12866_2018_1214_MOESM3_ESM.docx]

**Table S2.** Saturated and monounsaturated fatty acids composition in Antarctic yeasts.

| Yeast species | **Saturated** |  |  |  |  |  |  |
| --- | --- | --- | --- | --- | --- | --- | --- |
|  | <C14:0 | Myristic acid C14:0 | C15:0 | Palmitic acid  C16:0 | C17:0 | Stearic acid C18:0 | >C18:0 |
| *L. creatinivorum* | 2.4 | 1.2 | 0.2 | 12.2 | 0.5 | 4.3 | 13.9 |
| *C. parapsilosis* | 22.4 | 2.9 | 0.5 | 15.2 | nd | 5.0 | nd |
| *G. victoriae* | 1.5 | 2.9 | 1.0 | 23.3 | nd | 7.0 | 1.6 |
| *D. fristingensis* | ND | ND | nd | 11.0 | nd | nd | nd |
| *R. mucilaginosa* | 0.2 | 0.5 | nd | 13.4 | nd | 1.4 | nd |
| *C. laryngis* | 0.3 | 1.2 | 0.3 | 15.2 | 0.3 | 2.1 | 0.8 |
| *S. salmonicolor* | 5.6 | 6.3 | nd | 24.7 | nd | 7.1 | nd |
| *W. anomalus* | 0.4 | 0.3 | 0.3 | 15.6 | 0.5 | 4.6 | 0.9 |
| *G. gastrica* | ND | 0.6 | nd | 11.0 | 0.3 | nd | 1.5 |
| *M. gelida* | 2.4 | 3.2 | nd | 34.6 | nd | 49.3 | nd |
| *M. blollopis* | 2.1 | 6.9 | 0.7 | 25.8 | nd | 8.6 | nd |

| **Monounsaturated** | <C18:1 | Oleic acid C18:1 | C19:1 | Eicosenoic acid C20:1 | C20:1< |
| --- | --- | --- | --- | --- | --- |
| *L. creatinivorum* | 61.3 | 41.5 | 0.1 | 0.5 | nd |
| *C. parapsilosis* | 0.9 | 19.1 | ND | nd | nd |
| *G. victoriae* | 1.6 | 28.1 | ND | nd | nd |
| *D. fristingensis* | nd | 57.1 | 3.9 | nd | nd |
| *R. mucilaginosa* | 0.9 | 57.4 | ND | 1.7 | nd |
| *C. laryngis* | 78.6 | 63.3 | ND | 2.1 | 0.7 |
| *S. salmonicolor* | nd | 41.9 | ND | nd | nd |
| *W. anomalus* | 78.8 | 23.5 | 0.1 | 8.4 | 23.7 |
| *G. gastrica* | 78.2 | 42.3 | nd | nd | nd |
| *M. gelida* | 2.0 | 38.1 | nd | nd | nd |
| *M. blollopis* | 2.0 | 27.7 | nd | nd | nd |

Nd, not detected. C_N:D_ “N” represents the number of carbon atoms of each FD. and “D” is the number of double bonds in the fatty acids.
